# The use of psychiatric medications and associated factors among people receiving care at a transgender outpatient clinic in Southern Brazil, 2021-2022

**DOI:** 10.1590/S2237-96222024v33e2024170.especial.en

**Published:** 2024-12-06

**Authors:** Bruna dos Passos Gimenes, Adalvane Nobres Damaceno, Andrei Fernandes da Rocha, Guilherme Lamperti Thomazi, Gabriela Tizianel Aguilar

**Affiliations:** 1 Universidade do Vale do Rio dos Sinos, Programa de Residência Integrada Multiprofissional, Porto Alegre, RS, Brazil; 2 Universidade do Vale do Rio dos Sinos, Programa de Residência Integrada Multiprofissional, São Leopoldo, RS, Brazil; 3 Ministério da Saúde, Departamento de Atenção Especializada e Temática, Brasília, DF, Brazil; 4 Universidade de São Paulo, Faculdade de Saúde Pública, Programa de Pós-Graduação em Saúde Pública, São Paulo, SP, Brazil; 5 Universidade do Vale do Rio dos Sinos, Faculdade de Medicina, Porto Alegre, RS, Brazil e Aids Healthcare Foundation Brasil, Porto Alegre, RS, Brazil

**Keywords:** Personas Transgénero, Minorías Sexuales y de Género, Salud Mental, Utilización de Medicamentos, Estudios Transversales, Transgender People, Sexual and Gender Minorities, Mental Health, Medication Use, Cross-Sectional Studies

## Abstract

**Objective:**

To investigate the prevalence of psychiatric medication use and sociodemographic factors, including gender identity, age, race/skin color, education level, formal employment, and access to Primary Health Care (PHC) centers, among individuals receiving care at a transgender outpatient clinic in Southern Brazil.

**Methods:**

**:** This was a cross-sectional study using administrative data from the information systems of the Municipal Health Department of Porto Alegre, the capital city of Rio Grande do Sul state, collected between 2021 and 2022.

**Results:**

**:** This study analyzed 629 records of individuals who accessed the outpatient clinic and found a 29% prevalence (95%CI 25;32) of psychiatric medication use, with the most frequent class being psychoanaleptics (45%), especially fluoxetine (31%).

**Conclusion:**

In addition to a trend toward mixed-race race/skin color and incomplete elementary school, transgender people aged 40 to 49 years and with access to a Primary Health Care center were more likely to use psychiatric medications.

## Study contributions

**Main results:**

Prevalence of 29% (95%CI 25;32) of psychiatric medication use was observed, with a higher likelihood among individuals aged 40 to 49 years, with access to primary healthcare centers. Low education level and mixed-race/skin color showed significant effect sizes. The most frequently dispensed drug class was psychoanaleptics (45%), with fluoxetine (31%) standing out.

**Implications for services:**

This study can be replicated in other transgender outpatient clinics across Brazil and should contribute to broadening the discussion on mental healthcare for the transgender population.

**Perspectives:**

The findings highlight the importance of interdisciplinary approaches to healthcare and underscore the need to assess the effectiveness of public mental health policies for the transgender population.

## RESUMEN

**Objetivo:**

Investigar la prevalencia del uso de medicamentos psiquiátricos y los factores sociodemográficos, incluyendo identidad de género, edad, color de piel, nivel educativo, empleo formal y acceso a unidades de Atención Primaria de Salud (APS), entre personas atendidas en un ambulatorio trans en el sur de Brasil.

**Métodos:**

Estudio transversal que utilizó datos administrativos de los sistemas de información de la Secretaría Municipal de Salud de Porto Alegre, recolectados entre 2021 y 2022.

**Resultados:**

Este estudio analizó 629 registros de personas que accedieron al ambulatorio, encontró una prevalencia del 29% (IC_95_%: 25-32) en el uso de medicamentos psiquiátricos, con mayor frecuencia en la clase psicoanaléptica (45%), destacando el uso de fluoxetina (31%).

**Conclusión:**

Además de la tendencia relacionada al color pardo y educación primaria incompleta, las personas trans de 40 a 49 años, con acceso a una Unidad de Atención Primaria de Salud, presentaron mayor probabilidad de usar medicamentos psiquiátricos.

## INTRODUCTION

Transgender people are those who do not identify with the sex/gender assigned to them at birth, and may identify as trans men, trans women, transvestites or non-binary, experiencing or not the man-woman gender binary.^1^ Estimates regarding the proportion of people who identify as transgender or non-binary in the adult population vary. In the United States, 1.3 million, or 0.6% of adults, identify as transgender,^2^ while in Brazil, there are 3 million, or 2% of the adult population.^3^

This population faces significant disparities in terms of quality of life and mental health when compared to the general population. These differences may be driven by stigma, discrimination and violence, which are motivated by prejudice, such as hate crimes based on sexual orientation or gender identity.^4^ According to a report published by the National Association of Transvestites and Transsexuals (*Associação Nacional de Travestis e Transexuais* – ANTRA), between 2017 and 2023, it is estimated that 1,057 transgender people and transvestites were murdered in Brazil, amounting to about 13 deaths per month. Most of these victims were trans women or transvestites, sex workers, of Black race/skin color, and with low levels of education, making Brazil the country with the highest number of trans murders worldwide for the fourteenth consecutive year.^5^

Furthermore, transgender people are considered a social minority due to historical processes of marginalization and discrimination. This results in various social disadvantages, such as minority stress, gender dysphoria, and difficulties in accessing basic rights like education, healthcare, employment, and legal recognition of gender identity. These challenges negatively impact biopsychosocial well-being and exacerbate health problems, particularly in mental health, as this population frequently experiences multiple forms of violence and prejudice, increasing the risk of mental disorders.^6,7^

Thus, this study aims to estimate the prevalence of psychiatric medication use and associated factors among people receiving care at a transgender outpatient clinic in a Brazilian capital city, in order to address collective mental health in the trans population.

## METHODS

This is an analytical, cross-sectional, epidemiological study that followed the guidelines of the Strengthening the Reporting of Observational Studies in Epidemiology. The study was conducted using secondary data from the Transgender Outpatient Clinic and the Medication Dispensing Information System (*Sistema de Informação de Dispensação de Medicamentos* – DIS). in the city of Porto Alegre. Data collection was carried out using the clinic’s administrative database and the DIS. From the former, sociodemographic information was retrieved, and from the latter, data on dispensed medications were collected.

Historically, the transsexualization process in Brazil, which focused on hospital and surgical procedures, did not address the general healthcare needs of transgender people. This process involved gender affirmation surgeries and other medical procedures in specialized centers, but faced significant challenges, such as the lack of a holistic approach that considered the comprehensive health of transgender people, resulting in a fragmented care. Until 2019, the year the Transgender Outpatient Clinic was established, there were only five registered services in the country.

In 2017, the creation of the LGBT Health Technical Area and the Operational Plan represented a significant advancement in addressing the needs of the transgender population. The Trans Outpatient Clinic, integrated into the 2019-2021 II Operational Plan of the Municipal Comprehensive Health Policy for Lesbians, Gays, Bisexuals, Transvestites, Transsexuals, Queers, Intersex and Non-Binary People (PMSILGBTQI+) in Porto Alegre, was established to meet the demands of the local trans and transvestite social movement since 2018. This service was an important step in constructing a more inclusive healthcare system tailored to the specific needs of this population.

The Trans Outpatient Clinic was inaugurated in 2019 as a fully funded service by the Brazilian National Health System (*Sistema Único de Saúde* – SUS) and operating on an “open door” basis. Initially, it was implemented without the necessary funding, specific staff or adequate infrastructure, a situation that persisted until the service was relocated, allowing for a reorganization and improvements in care. Following the relocation, the outpatient clinic began offering individual and group consultations, as well as medical and nursing procedures. Aimed at transgender people and transvestites living in Porto Alegre, its services are equivalent to those provided by a primary health care (PHC) center, offering comprehensive and inclusive care. In addition, it is serves as a field of practice for multi-professional residency programs in mental health, family and community health, and public health.

This context highlights the importance of health policies that address the specific needs of the transgender population, integrating specialized care with a broad and inclusive approach. While national policies are essential, their local implementation is crucial in transforming reality. Although national policies provide framework for dialogue, local initiatives, such as the Transgender Outpatient Clinic, are key to significant progress and more effective, accessible care for transgender people.^8^

The DIS is an electronic system implemented in Porto Alegre to manage the medication dispensing, containing general identification information about users, such as name, SUS card, date of birth, mother’s name, and taxpayer identification number (*Cadastro de Pessoa Física* – CPF). At the time of data collection, information on race/skin color, gender identity, or sexual orientation was not included.

To collect outcome data, the registry from the Transgender Outpatient Clinic was used to perform individual searches in the DIS system using the SUS card number and/or CPF. Additionally, the 2023 Municipal List of Essential Medicines (*Relação Municipal de Medicamentos Essenciais* – REMUME) was used to classify and identify medications subject to special control.

### Population and data analysis

A total of 629 records of people who accessed the Transgender Outpatient Clinic between 2021 and 2022, were aged 18 years or older and resided in the municipality. The study outcome considered the use of psychiatric medication as measured by the dispensing recorded in the DIS. Independent variables included sociodemographic characteristics and healthcare access, such as gender identity, age, race/skin color, education level, formal employment and access to a PHC center.

Statistical analysis was performed using the Statistical Package for the Social Sciences (SPSS®), version 18.0. For the analysis of sample distribution and prevalence of psychiatric medication use according to independent variables, the Mann-Whitney U test, Pearson’s chi-square test, and Fisher’s exact test were used, with a significant level of 20%. To estimate the crude and adjusted prevalence ratios (PR) of psychiatric medication use based on sociodemographic variables, a backward Poisson regression approach was used, with robust variance, with a 5% significance level. Medications were classified according to their pharmacological class, based on the Anatomical Therapeutic Chemical (ATC).

### Ethical considerations

The project was submitted to the Research Ethics Committee of the Municipal Health Department of Porto Alegre, Opinion No. 6,460,330.

As this study used secondary data, the Free and Informed Consent Form was replaced by the data confidentiality agreement regarding data handling.

## RESULTS

A total of 263 (41.9%) trans men, 227 (36.1%) trans women, 101 (16.1%) non-binary people, 16 (2.6%) transvestites and 21 (3.3%) individuals categorized as “other”, as they did not identify with any of the gender categories presented when they first started receiving care at the service. Regarding race/skin color, 436 (72.7%) individuals self-identified as White; 69 (11.5%), mixed-race; 86 (14.3%), Black; 2 (0.3%), Indigenous; and 7 (1.2%), Asian. As for age, the median age was 26 years [IQ 22; 32], with a minimum age of 18 years and a maximum of 71 years. With regard to education, 29 (5.2%) people had incomplete elementary school; 20 (3.6%), had complete elementary school; 14 (18.7%), incomplete high school; 191 (34.4%), complete high school; 142 (25.6%), incomplete higher education; and 69 (12.4%), complete higher education.

Regarding social name rectification, 118 (19.1%) people had their name legally changed and 499 (80.9%) had not rectified their name. Furthermore, regarding formal employment, 182 (34%) individuals reported having a formal job and 354 (66%) were not formally employed. Concerning access to PHC centers, 298 (47.4%) individuals reported attending their reference healthcare center, while 330 (52.6%) had not accessed it.

The prevalence of psychiatric medication use of 29% (95%CI 25;32) was observed among people registered at the Transgender Outpatient Clinic. Significant frequencies for psychiatric medication use were observed among individuals with a median age of 27 years [IQR 23; 38], those identifying as mixed-race (44%), those with incomplete elementary school (45%), and those who had accessed a PHC center (40%), as shown in Table 1. 

**Table 1:** Sample distribution and prevalence of psychiatric medication use according to independent variables among people at the Transgender Outpatient Clinic, Porto Alegre, Rio Grande do Sul state, Brazil, 2021-2022 (n = 629)

Variable	n (%)/median (IQR)	**Use of psychiatric medications (%)**		p-value
		**Absence**	**Presence**	
**Age**	**26 (IQ 22;32)**	**25 (IQ 22;30)**	**27 (IQ 23;38)**	**< 0.001** ^a^
Gender identity				
Trans man	263 (41,9)	195 (74,1)	68 (25,9)	0.420^b^
Trans woman	227 (36,1)	159 (70)	68 (30)	
Non-binary	101 (16,1)	72 (71,3)	29 (28,7)	
Transvestite	16 (2,6)	9 (56,2)	7 (43,8)	
Other	21 (3,3)	13 (61,9)	8 (38,1)	
**Race/skin color**				
White	436 (72,7)	318 (72,9)	118 (27,1)	0.040^c^
Mixed-race	69 (11,5)	39 (56,5)	30 (43,5)	
Black	86 (14,3)	58 (67,4)	28 (32,6)	
Indigenous	2 (0,3)	1 (50)	1 (50)	
Yellow	7 (1,2)	6 (85,7)	1 (14,3)	
**Education level**				
Completed higher education	69 (12,4)	55 (79,7)	14 (20,3)	0.091^b^
Incomplete higher education	142 (25,6)	103 (72,5)	39 (27,5)	
Complete high school	191 (34,4)	137 (71,7)	54 (28,3)	
Incomplete high school	1 04 (18,7)	78 (75)	26 (25)	
Completed elementary school	20 (3,6)	11 (55)	9 (45)	
Incomplete elementary school school	29 (5,2)	16 (55,2)	13 (44,8)	
**Social name rectification**				
Yes	118 (19,1)	84 (71,2)	34 (28,8)	0.869^b^
No	499 (80,9)	359 (71,9)	140 (28,1)	
**Formal work**				
Yes	182 (34)	134 (73,6)	48 (26,4)	0.387^b^
No	354 (66)	248 (70,1)	106 (29,9)	
**Access** **S** **to a healthcare center**				
Yes	298 (47,4)	178 (59,7)	120 (40,3)	<0.001^b^
No	330 (52,6)	270 (81,8)	60 (18,2)	

a) Mann-Whitney U test; b) Pearson’s chi-square test; c) Fisher’s exact test.

A total of 391 psychiatric medication dispensations were performed during the study period, with the highest proportion dispensed for the psychotropic category (45%), notably fluoxetine (31%), followed by psycholeptic category (36%), with chlorpromazine being the most common (12%). On the other hand, the lowest frequencies were observed in the antiepileptic and anti-parkinsonian categories, with 17% and 1%, respectively, valproic acid (9%) being the most used antiepileptic treatment (Table 2).

**Table 2:** Absolute and relative frequency of medications, according to the Anatomical Therapeutic Chemical classification, dispensed through the Medication Dispensing Information System, Porto Alegre, Rio Grande do Sul state, Brazil, 2021 2022 (n = 391)

Class	n	%
**Antiepileptic**		
Valproic acid	34	8.7
Clonazepam	23	5.9
Carbamazepine	10	2.6
Phenytoin	1	0.3
Phenobarbital	-	-
Total	68	17.4
**Anti-parkinsonian**		
Biperiden	5	1
Total	5	1
**Psycholeptics**		
Chlorpromazine	48	12.3
Lithium carbonate	47	12.0
Diazepam	23	5.9
Haloperidol	22	5.6
Total	140	35.8
**Psychoanaleptic**		
Fluoxetine	123	31.5
Amitriptyline	42	10.7
Imipramine	10	2.6
Nortriptyline	3	0.8
Total	178	45.5
**Grand total**	391	100

As for the multivariate analysis of the data, the results of the effect between sociodemographic and access factors with the use of psychiatric medication in people receiving care at the Transgender Outpatient Clinic indicate that, after adjusting for variables using the elimination method, the factors “age group” and “access to a healthcare center” were associated with a higher likelihood of psychiatric medication use. Therefore, it could be seen that people aged 40 to 49 years were 2,06 (IC_95_% 1,55;2,73) times more likely to use psychiatric medications when compared to younger age groups. On the other hand, the lack of access to a PHC center showed a protective factor of 0.46 (95%CI 0.35;0.60), when compared to those with access. Although the variables “education level” and “race/skin color” lost statistical significance in the adjusted model, they showed an association trend in the bivariate analysis (Table 3). Individuals with incomplete elementary school showed the highest adjusted PR, of 1.39 (95%CI 0.91;2.12), and those identifying as mixed-race had the highest adjusted PR of 1.33 (95%CI 0.97;1.81). The “Indigenous” category was not included in the multivariable analysis due to equal distribution (50%) between groups, preventing precise estimation of statistical effects.

**Table 3:** Crude and adjusted prevalence ratios (PR) with 95% confidence intervals (95%CI), of psychiatric medication use, according to independent variables, in people receiving care at the Transgender Outpatient Clinic, Porto Alegre, Rio Grande do Sul state, Brazil, 2021-2022 (n = 629)

Variable	Gross PR (95%CI)	PR adjusted (95%CI)
Gender identity		
Trans man	1	1
Trans woman and transvestite	1.19 (0.90;1.57)	1.04 (0.76;1.40)
Non-binary	1.11 (0.76;1.60)	1.23 (0.82;1.86)
Others	1.47 (0.82;2.63)	1.05 (0.55;1.99)
**Age group (years)**		
18-23	1	1
24-29	0.99 (0.71;1.40)	0.96 (0.68;1.35)
30-39	1.15 (0.80;1.63)	1.10 (0.78;1.55)
40-49	2.37 (1.76;3.19)	2.06 (1.55;2.73)
50+	1.36 (0.59;3.11)	1.58 (0.78;3.19)
**Education**		
Complete high school or higher education	1	1
Incomplete high school	1.06 (0.76;1.46)	1.11 (0.80;1.54)
Incomplete elementary school	1.68 (1.08;2.60)	1.39 (0.91;2.12)
**Race/skin color**		
White	1	1
Mixed-race	1,61 (1,18;2,19)	1.33 (0.97;1.81)
Black	1,21 (0,86;1,69)	1.02 (0.72;1.45)
Asian	0,53 (0,09;3,27)	0.49 (0.09;2.62)
**Social name rectification**		
Yes	1	1
No	0.97 (0.70;1.33)	1.05 (0.76;1.45)
**Formal work**		
Yes	1	1
No	1.13 (0.84;1.51)	1.12 (0.83;1.52)
**Access to a healthcare center**		
Yes	1	1
No	0.45 (0.34;0.59)	0.46 (0.35;0.60)

## DISCUSSION

This study presented results that showed a prevalence of psychiatric medication dispensing in 29% of the sample. Being a person of Black race/skin color and having incomplete elementary school were observed as risk factors in the bivariate analysis. Furthermore, characteristics such as being an adult and having access to a PHC center remained associated in the adjusted model.

It is worth highlighting that the lack of official data on transgender people and transvestites in Brazil, a serious issue that hampers the implementation and improvement of public policies. The Census and the National Health Policy (*Política Nacional de Saúde* – PNS) do not contain fields that refer to gender identity. As such, the data available in Brazil are primarily obtained from academic research and surveys conducted by non-governmental organizations led by transgender people, such as ANTRA.^5^

According to the World Health Organization, the global prevalence of mental disorders in the population is 13%; however, this occurrence may vary based on different population characteristics.^9^ In Brazil, no comprehensive estimate for all common mental disorders has been found in the literature. However, according to the 2019 National Health Survey, approximately 10% of the population reported having received a diagnosis of depression from a mental health professional.^10^ Regarding the use of psychiatric medications, we found only one systematic review addressing the Brazilian population. However, this study is limited to the prevalence of the use of a specific class of drugs – antidepressants – and reported an overall proportion of 4% across the studies reviewed.^11^ In our study, it could be seen that the frequency for fluoxetine was 31.5%, and for amitriptyline was 10.7%. Taking into consideration that transgender people and transvestites are more exposed to various types of violence and experience greater social vulnerability, they are at higher risk of developing mental health problems compared to the general population. This risk is still evident among transgender people, and varies depending on factors such as age, race/skin color, place of residence, education level and economic situation.^12,13^

Regarding the median age in the study for dispensing psychiatric medication, it could be seen that older people had a higher prevalence of medication withdrawal, and those aged 40 to 49 years had the highest adjusted PR. This phenomenon can be partially explained by the fact that these individuals grew up and lived in a society that considered sexual orientation and gender identity as mental disorders.^14^ Thus, only in 2019, transgender was removed from the category of mental health disorders and included in the category of conditions related to sexual health, in the International Classification of Diseases. Studies have shown that the depathologization of transgender identity, gender affirmation processes, the use of the social name and self-declared gender were associated with the reduction of symptoms of anxiety and depression, leading to a sense of belonging, improved social integration, greater engagement of transgender people to healthcare services and improved quality of life.^15,16^

The dispensing of psychiatric medications was more prevalent among individuals self-identified as Black and mixed-race with lower levels of education, despite greater access to outpatient services being observed among. White individuals with higher level of education. Given that, studies show that self-declared Black transgender people, who face more discrimination and violence, have a lower level of education, are more likely to be employed in underpaid jobs, experience worse socioeconomic conditions and have lower life expectancy, resulting in a significantly higher risk of developing health problems and psychological distress.^17,18^ According to a survey conducted by Antra , entre 2017 e 2023, approximately 17 78,7% of transgender people murdered were Black, and 57% were sex workers.^5^ Given that, the concept of intersectionality allows us to better understand this result, as it analyzes the multiple forms of discrimination that a person can experience, which more pronounced among transgender, Black people and people belonging to social classes with lower purchasing power.^18^

Regarding healthcare access, recognizing the vulnerability of the transgender population and its historical detachment from the SUS, the PNSILGBT was launched to ensure universal access to healthcare for this population. It establishes guidelines for inclusive care and comprehensive prevention and health promotion. However, when attempting to access healthcare services, transgender people face several barriers, such as the pathologization of their identities, institutional prejudice and discrimination by healthcare professionals, resistance to the use of their social name and appropriate pronouns, and lack of support.^19,20^ These barriers contribute to delays in seeking preventive care, increasing the risk of developing chronic diseases and worsening mental health problems.

Historical discrimination imposed by heteronormative, sexist, racist and classist society, can lead to social and institutional marginalization of the transgender population, contributing to increasing the risk of developing mental health problems, high-risk behavior for sexually transmitted infections and substance abuse.^4,6,7^ In this study, the medications most frequently dispensed included fluoxetine, followed by chlorpromazine, lithium carbonate, amitriptyline and valproic acid. Although we present the frequency of medications dispensing according to the ATC classification, we use the perspective of the therapeutic approach and recommendation of the use of medications in our discussion, to expand the understanding of their use in mental health care.

Overall, studies indicate high prevalence of mental disorders, such as depression, anxiety and suicide behavior among transgender people, as a result of daily transphobic violence.^21^ The data presented in this study corroborate the scientific literature regarding the mental suffering of this population, with approximately 70% of the 659 people in the study diagnosed with depression and anxiety, and a significant portion reporting suicidal thoughts.^22^ In addition, they were more likely to be prescribed for antidepressants and anxiolytics and to be hospitalized following suicide attempts.^23^ In our study, we observed prevalence in the dispensing of fluoxetine, amitriptyline, clonazepam and diazepam, medications typically used in the treatment of depression and anxiety, contributing to reducing symptoms.

The mental health disparities among transgender people can be associated with minority stress, as both daily stressors and specific stressors negatively impact mental health indicators and quality of life of this population.^24^ A study conducted in Chicago, which used a population cohort of young people from sexual and gender minorities, associated family rejection, among transgender people, with increased symptoms of depression and social isolation associated with suicidal ideation. This study also compared social, family and friend support, between transgender youth and minority cisgender youth, identifying lower levels of support among transgender youth in their relationships.^25^ This finding is in line with other studies that found a strong correlation between stigmatization, discrimination and severe depression.^26^

The literature also suggests that the excessive burden of social stressors associated with low levels of education, informal access to the labor market, and fragile social support is linked to early use of psychoactive substances and a higher risk of substance abuse.^27,28^ Furthermore, it is worth highlighting that mental suffering is not only due to gender identity, but rather arises from the intersection between gender, race, territory, and educational level, with specific segments of this population (Black and impoverished people) being exposed to greater survival risks.^5^

In our study, the results of the PR on the use of psychiatric medication contribute to the discussion in the field of health policies aimed at the transgender population. It should be noted that, despite the efforts represented by the implementation of the PNSILGBT, there remains a challenge in ensuring that this population has equitable access to health care. Given the results of this study, it is clear that inequalities in access to health services intensify the historical vulnerabilities and disparities experienced by these people. Transgender people do not suffer solely because they are transgender, but because of society’s lack of acceptance and the prejudice they encounter, which negatively impacts various aspects of their lives, such as education, employment, relationships, and health.

It is essential to recognize that, when administered safely and appropriately, medication can control and alleviate the symptoms of mental disorders. However, psychiatric medication use remains stigmatized due to misconceptions about mental disorders and their treatment. The decision to prescribe psychotropic medications must consider the patient’s diagnosis, medical history, age, comorbidities, and the concurrent use of other medications or substances. On the one hand, we discuss whether the prevalence of psychiatric medication use may be an intermediary for the high occurrence of mental disorders among the transgender population; on the other hand, we acknowledge the importance of equity and guaranteeing access to pharmacological technologies.

Access to medications should be viewed as an essential part of mental health care, particularly given the numerous barriers transgender individuals face when accessing healthcare services. The lack of mental health services within the SUS exacerbates this situation, resulting in limited access to psychiatric medications and psychotherapies for this population. Therefore, improving safe and adequate access to medications is crucial to addressing the mental health needs of these vulnerable groups.

Furthermore, when accessing health services, transgender people face complex and multifaceted organizational barriers related to discrimination, pathologization, inadequate reception and sociodemographic factors, which result in healthcare avoidance.^29^ It is important to recognize that a successful model of equitable and comprehensive healthcare requires acknowledging the specific needs of each group. A strategy to overcome these barriers and fully implement the policy includes the training of healthcare professionals and support staff to improve the reception and care for this population.^30^

The limitations of this study include its cross-sectional design, which hinders inferences about changes in exposure over time. Additionally, the scarcity of studies addressing medication use among the transgender population highlights the need for further research on this topic so that effective public policies can be restructured, recognizing that the high prevalence of medication use may reflect mental suffering due to health inequities. We believe that the frequency of medication use was underestimated, as we relied solely on dispensing data as a secondary collection instrument. Therefore, the use of psychometric scales through validated questionnaires should be encouraged. Another limiting factor is the issue of access to treatment and, consequently, to medications, which may have contributed to underestimating the problem. 

This study estimated the prevalence of psychiatric medication dispensing and associated factors among individuals receiving care at the Transgender Outpatient Clinic in a capital city in Southern Brazil. The results show that 29% of individuals registered with the service collected one or more medications used to treat mental disorders, with age and lack of access to a PHC center being factors associated with the outcome. Although education and race/skin color, did not remain in the adjusted model, they showed moderate effect measures in the adjusted analysis. Based on these findings, we discuss how stigma and discrimination are vulnerability factors that expose the transgender population to high levels of mental suffering, which is increased when associated with gender, race/skin color, education, and social class.
