# Supplementary material for: The use of psychiatric medications and associated factors among people receiving care at a transgender outpatient clinic in Southern Brazil, 2021-2022
Source: Epidemiol Serv Saude. 2024 Dec 6;33(spe1):e2024170. doi: 10.1590/S2237-96222024v33e2024170.especial.en (PMC11654390; doi:10.1590/S2237-96222024v33e2024170.especial.en)
Supplement: Translation

# Translation: Uso de medicamentos psiquiátricos e fatores associados em pessoas atendidas em um ambulatório trans no Sul do Brasil, 2021-2022

**DOI:** 10.1590/S2237-96222024v33e2024170.especial.pt

**Authors:** Bruna dos Passos Gimenes, Adalvane Nobres Damaceno, Andrei Fernandes da Rocha, Guilherme Lamperti Thomazi, Gabriela Tizianel Aguilar

**Affiliations:** 1 Universidade do Vale do Rio dos Sinos, Programa de Residência Integrada Multiprofissional, Porto Alegre, RS, Brasil; 2 Universidade do Vale do Rio dos Sinos, Programa de Residência Integrada Multiprofissional, São Leopoldo, RS, Brasil; 3 Ministério da Saúde, Departamento de Atenção Especializada e Temática, Brasília, DF, Brasil; 4 Universidade de São Paulo, Faculdade de Saúde Pública, Programa de Pós-Graduação em Saúde Pública, São Paulo, SP, Brasil; 5 Universidade do Vale do Rio dos Sinos, Faculdade de Medicina, Porto Alegre, RS, Brasil e Aids Healthcare Foundation Brasil, Porto Alegre, RS, Brasil

**Keywords:** Pessoas Transgênero, Minorias Sexuais e de Gênero, Saúde Mental, Uso de Medicamentos, Estudos Transversais

## Abstract

**Objetivo:**

Investigar a prevalência do uso de medicamentos psiquiátricos e fatores sociodemográficos, incluindo identidade de gênero, idade, raça/cor da pele, escolaridade, trabalho formal e acesso a unidades de saúde da atenção primária à saúde (APS), em pessoas atendidas em um ambulatório trans, no Sul do Brasil.

**Métodos:**

Estudo do tipo transversal, que utilizou dados administrativos de sistemas de informação da Secretaria Municipal de Saúde de Porto Alegre, coletados entre 2021 e 2022.

**Resultados:**

Este estudo analisou 629 registros de pessoas que acessaram o ambulatório e encontrou uma prevalência de 29% (IC_95_% 25;32) de uso de medicamentos psiquiátricos, com maior frequência para a classe de psiconaléptico (45%), com destaque para a medicação fluoxetina (31%).

**Conclusão:**

Além da tendência para raça/cor parda e ensino fundamental incompleto, pessoas trans de 40 a 49 anos e com acesso a unidade de APS apresentaram maior probabilidade de utilizar medicamentos psiquiátricos.

## Contribuições do estudo

**Principais resultados:**

Observou-se uma prevalência de uso de medicamentos psiquiátricos de 29% (IC_95_% 25;32), com maior probabilidade para pessoas de 40 a 49 anos e com acesso a unidades de saúde da atenção primária. Baixa escolaridade e raça/cor parda apresentaram tamanhos de efeito importantes. A classe mais dispensada foi a de psicoestimulantes (45%), com destaque para a fluoxetina (31%).

**Implicações para os serviços:**

Este estudo pode ser reproduzido em outros ambulatórios trans do Brasil e deve contribuir para ampliar a discussão do cuidado em saúde mental da população trans

**Perspectivas:**

Os achados ressaltam a importância de se pensar o cuidado em saúde de forma interdisciplinar e se discutir a efetividade das políticas públicas em saúde mental para a população trans.

## INTRODUÇÃO 

As pessoas trans são aquelas que não se identificam com o sexo/gênero que lhes foi socialmente atribuído ao nascimento, podendo se identificar como homem trans, mulher trans, travesti ou não binárias, vivenciando ou não a binaridade homem ou mulher.^1^ As estimativas sobre a proporção de pessoas que se identificam como transgênero ou não binário na população adulta variam. Nos Estados Unidos, 1,3 milhão, ou 0,6% dos adultos, se identificam como transexuais,^2^ e no Brasil, são 3 milhões, ou 2% da população adulta do país.^3^

Essa população enfrenta significativas disparidades em relação à qualidade de vida e à saúde mental, quando comparada à população em geral. Tais diferenças podem ser motivadas pelo estigma, pela discriminação e pela violência, que são motivadas por preconceitos, como os crimes de ódio baseados na orientação sexual ou identidade de gênero.^4^ Segundo relatório divulgado pela Associação Nacional de Travestis e Transexuais (Antra), entre os anos de 2017 e 2023, estima-se que 1.057 pessoas trans e travestis foram assassinadas no Brasil, ou seja, cerca de 13 mortes por mês. A maioria dessas vítimas eram mulheres trans ou travestis, profissionais do sexo, negras e de baixa escolaridade, sendo o Brasil o país que mais mata pessoas trans no mundo, pelo décimo quarto ano consecutivo.^5^

Além disso, pessoas trans são consideradas minorias sociais devido a processos históricos de marginalização e discriminação. Isso resulta em diversos prejuízos sociais, como o estresse de minorias, disforia de gênero e dificuldade de acesso a direitos básicos, como educação, saúde, emprego e reconhecimento legal da identidade de gênero. Essas dificuldades influenciam negativamente o bem-estar biopsicossocial e agravam problemas de saúde, especialmente na saúde mental, pois essa população é frequentemente vítima de diversas formas de violência e preconceito, aumentando o risco de transtornos mentais.^6,7^

Desse modo, este estudo tem como objetivo estimar a prevalência de uso de medicamentos psiquiátricos e fatores associados em pessoas atendidas em um ambulatório trans de uma capital brasileira, a fim de discutir o tema da saúde mental coletiva na população trans.

## MÉTODOS 

Trata-se de estudo epidemiológico, do tipo transversal, analítico e que utilizou as diretrizes do *Strengthening the Reporting of Observational Studies in Epidemiology*. O estudo foi desenvolvido com dados secundários do Ambulatório Trans e do Sistema de Informação de Dispensação de Medicamentos (DIS) do município de Porto Alegre. Para coleta de dados, foi utilizado o banco administrativo do serviço e o DIS. Do primeiro, foram recuperadas informações sociodemográficas; e do segundo, os medicamentos dispensados.

O processo transexualizador no Brasil, com foco hospitalar e cirúrgico, historicamente não atendia às necessidades gerais das pessoas trans. Esse processo envolvia a realização de cirurgias de redesignação sexual e outros procedimentos médicos em centros especializados, mas enfrentava desafios significativos, como a ausência de uma abordagem holística que considerasse a saúde integral da pessoa trans, resultando em uma abordagem fragmentada do cuidado. Até 2019, ano da abertura do Ambulatório Trans, havia apenas cinco serviços cadastrados no país.

Em 2017, a criação da Área Técnica da Saúde LGBT e do Plano Operativo representou um avanço significativo na abordagem das necessidades da população trans. O Ambulatório Trans, integrado ao II Plano Operativo 2019-2021 da Política Municipal de Saúde Integral de Lésbicas, Gays, Bissexuais, Travestis, Transexuais, Queers, Intersexuais e Pessoas com Identidade de Gênero Não Binária (PMSILGBTQI+) de Porto Alegre, foi estabelecido para atender às demandas do movimento social trans e travesti da cidade desde 2018. Este serviço foi um passo importante na construção de um sistema de saúde mais inclusivo e adaptado às necessidades específicas dessa população.

O Ambulatório Trans foi inaugurado em 2019, como um serviço totalmente financiado pelo Sistema Único de Saúde (SUS) e de modalidade “porta aberta”, inicialmente sendo realizado sem o fomento necessário, equipe específica ou estrutura adequada, persistindo até a transferência do serviço para um novo local, o que permitiu uma reorganização e melhorias no atendimento. Após a mudança, passou a oferecer atendimentos individuais e em grupo, além de procedimentos médicos e de enfermagem. Destinado a pessoas trans e travestis residentes em Porto Alegre, seu atendimento equivale ao de uma unidade de saúde da atenção primária à saúde (APS), proporcionando cuidados integrais e inclusivos. Além disso, é um campo de práticas para programas de residência multiprofissional em saúde mental, saúde da família e comunidade, e saúde coletiva.

Esse contexto evidencia a importância de políticas de saúde que atendam às necessidades específicas da população trans, integrando cuidados especializados com uma abordagem ampla e inclusiva. A existência de políticas nacionais é essencial, mas sua implementação local é crucial para transformar a realidade. Embora políticas nacionais tenham abertura para o diálogo, são as iniciativas locais, como o Ambulatório Trans, que realmente possibilitam avanços significativos e cuidados mais eficazes e acessíveis para pessoas trans.^8^

Por sua vez, o DIS é um sistema informatizado implementado no município de Porto Alegre para controle de dispensação de medicamentos, contendo informações gerais de identificação das pessoas usuárias, como nome, cartão SUS, data de nascimento, nome da mãe e o Cadastro de Pessoa Física (CPF). No momento da coleta de dados, não constavam informações sobre raça/cor da pele, identidade de gênero nem orientação sexual.

Dessa forma, para coletar os dados de desfecho, foi utilizada a lista de cadastros do Ambulatório Trans para se fazerem consultas individuais no DIS, através do número do cartão SUS e/ou do CPF. Além disso, considerou-se a Relação Municipal de Medicamentos Essenciais 2023 (Remume) para a classificação e identificação dos medicamentos de controle especial.

### População e análise de dados

Foram incluídos 629 registros de pessoas que acessaram o Ambulatório Trans entre os anos de 2021 e 2022, maiores de 18 anos e residentes no município. O desfecho do estudo considerou o uso de medicamento psiquiátrico medido pela dispensação registrada no DIS. As variáveis independentes foram as sociodemográficas e de acesso, e incluíram identidade de gênero, idade, raça/cor da pele, escolaridade, trabalho formal e acesso a unidade da APS. 

A análise estatística foi realizada com uso do programa Statistical Package for the Social Science (SPSS®), versão 18.0. Para análise da distribuição da amostra e prevalências de uso de medicação psiquiátrica de acordo com variáveis independentes, utilizou-se o Teste U de Mann-Whitney, o teste do qui-quadrado de Pearson e o teste Exato de Fisher, adotando-se um nível de significância de 20%. Para estimar a razão de prevalência (RP) bruta e ajustada do uso de medicação psiquiátrica conforme variáveis sociodemográficas, foi utilizado procedimento *backward* da regressão de Poisson, com variância robusta, adotando-se um nível de significância de 5%. Foi realizada a classificação dos medicamentos, conforme sua classe farmacológica, segundo a Anatomical Therapeutic Chemical (ATC). 

### Considerações éticas 

O projeto foi submetido ao Comitê de Ética em Pesquisa da Secretaria Municipal de Saúde de Porto Alegre, Parecer n^o^ 6.460.330.

Por tratar-se de dados secundários, o Termo de Consentimento Livre e Esclarecido foi substituído pelo termo de confidencialidade dos dados.

## RESULTADOS 

Foi observado um total de 263 (41,9%) homens trans, 227 (36,1%) mulheres trans, 101 (16,1%) pessoas não binárias, 16 (2,6%) travestis e 21 (3,3%) pessoas categorizadas como “outra”, uma vez que não se identificaram com nenhuma das categorias de gênero apresentadas quando iniciaram o acompanhamento no serviço. Quanto ao quesito raça/cor da pele, 436 (72,7%) pessoas se autodeclararam brancas; 69 (11,5%), pardas; 86 (14,3%), pretas; 2 (0,3%), indígenas; e 7 (1,2%), amarelas. Em relação à idade, foi observada uma mediana de idade de 26 anos [IQ 22; 32], sendo a idade mínima 18 anos e a máxima de 71 anos. No que se refere à escolaridade, 29 (5,2%) pessoas tinham ensino fundamental incompleto; 20 (3,6%), ensino fundamental completo; 14 (18,7%), ensino médio incompleto; 191 (34,4%), ensino médio completo; 142 (25,6%), ensino superior incompleto; e 69 (12,4%), ensino superior completo. 

No que tange à retificação do nome social, 118 (19,1%) pessoas tinham seu nome retificado e 499 (80,9%) não haviam retificado o nome. Ademais, no que se refere ao trabalho formal, 182 (34%) pessoas informaram estar em um trabalho formal e 354 (66%) não estariam em uma condição formal de trabalho. Quanto ao acesso a unidades da APS, 298 (47,4%) das pessoas informaram frequentar sua unidade de saúde de referência, e 330 (52,6%) não teriam acessado. 

Observou-se uma prevalência do uso de medicamentos psiquiátricos em 29% (IC_95_% 25;32) das pessoas registradas no Ambulatório Trans. As frequências significativas para uso de medicamento psiquiátrico foram observadas em pessoas com mediana de 27 anos de idade [IQ 23; 38], de raça/cor da pele parda (44%), com ensino fundamental incompleto (45%) e que acessaram unidade da APS (40%), conforme a tabela 1. 

**Tabela 1:** Distribuição da amostra e prevalências de uso de medicamento psiquiátrico de acordo com variáveis independentes em pessoas do Ambulatório Trans, Porto Alegre, 2021-2022 (n = 629)

Variável	n (%)/mediana (IQR)	Uso de medicamentos psiquiátricos (%)		p-valor
		**Ausência**	**Presença**	
**Idade**	26 (IQ 22;32)	25 (IQ 22;30)	27 (IQ 23;38)	< 0.001^a^
**Identidade de gênero**				
Homem trans	263 (41,9)	195 (74,1)	68 (25,9)	0.420^b^
Mulher trans	227 (36,1)	159 (70)	68 (30)	
Não binário	101 (16,1)	72 (71,3)	29 (28,7)	
Travesti	16 (2,6)	9 (56,2)	7 (43,8)	
Outro	21 (3,3)	13 (61,9)	8 (38,1)	
**Raça/cor da pele**				
Branca	436 (72,7)	318 (72,9)	118 (27,1)	0.040^c^
Parda	69 (11,5)	39 (56,5)	30 (43,5)	
Preta	86 (14,3)	58 (67,4)	28 (32,6)	
Indígena	2 (0,3)	1 (50)	1 (50)	
Amarela	7 (1,2)	6 (85,7)	1 (14,3)	
**Escolaridade**				
Superior completo	69 (12,4)	55 (79,7)	14 (20,3)	0.091^b^
Superior incompleto	142 (25,6)	103 (72,5)	39 (27,5)	
Médio completo	191 (34,4)	137 (71,7)	54 (28,3)	
Médio incompleto	104 (18,7)	78 (75)	26 (25)	
Fundamental completo	20 (3,6)	11 (55)	9 (45)	
Fundamental incompleto	29 (5,2)	16 (55,2)	13 (44,8)	
**Retificação do nome social**				
Sim	118 (19,1)	84 (71,2)	34 (28,8)	0.869^b^
Não	499 (80,9)	359 (71,9)	140 (28,1)	
**Trabalho formal**				
Sim	182 (34)	134 (73,6)	48 (26,4)	0.387^b^
Não	354 (66)	248 (70,1)	106 (29,9)	
**Acesso a unidade de sSaúde**				
Sim	298 (47,4)	178 (59,7)	120 (40,3)	<0.001^b^
Não	330 (52,6)	270 (81,8)	60 (18,2)	

a) Teste U de Mann-Whitney; b) Teste do qui-quadrado de Pearson; c) Teste exato de Fisher.

Foram realizadas um total de 391 dispensações de medicamentos psiquiátricos no período do estudo, tendo sido a maior proporção dispensada para a classe de psiconaléptico (45%), com destaque para a medicação fluoxetina (31%), seguida da classe de psicolépticos (36%), com destaque para a clorpromazina (12%). Por outro lado, as menores frequências observadas foram para as classes antiepilético e anti-parkinsoniano, com 17% e 1%, respectivamente, com ênfase no uso de ácido valpróico (9%) para tratamento antiepilético (Tabela 2). 

**Tabela 2:** Frequência absoluta e relativa de medicamentos, segundo a classificação Anatomical Therapeutic Chemical, dispensados no Sistema de Informação de Dispensação de Medicamentos, Porto Alegre, 2021 2022 (n = 391)

Classe	n	%
**Antiepilético**		
Ácido valpróico	34	8,7
Clonazepam	23	5,9
Carbamazepina	10	2,6
Fenitoína	1	0,3
Fenobarbital	-	-
Total	68	17,4
**Anti-parkinsoniano**		
Biperideno	5	1
Total	5	1
**Psicolépticos**		
Clorpromazina	48	12,3
Carbonato de lítio	47	12,0
Diazepam	23	5,9
Haloperidol	22	5,6
Total	140	35,8
**Psiconaléptico**		
Fluoxetina	123	31,5
Amitriptilina	42	10,7
Imipramina	10	2,6
Nortriptilina	3	0,8
Total	178	45,5
**Total geral**	391	100

Na análise multivariável dos dados, os resultados do efeito entre fatores sociodemográficos e de acesso para o uso de medicação psiquiátrica, em pessoas admitidas no Ambulatório Trans, apontam que, após o ajuste de variáveis por eliminação, os fatores “faixa etária” e “acesso a unidade de saúde” estão associados à maior probabilidade de utilizar medicamentos psiquiátricos. Portanto, foi observado que pessoas na faixa etária entre 40 e 49 anos apresentaram 2,06 (IC_95_% 1,55;2,73) vezes maior probabilidade de utilizar medicamentos psiquiátricos, quando comparada a faixas de idade menores. Por outro lado, a falta de acesso a unidade de saúde da APS mostrou fator de proteção em 0.46 (IC_95_% 0.35;0.60), quando comparado à presença do acesso. Embora as variáveis “escolaridade” e “raça/cor da pele” tenham perdido significância no modelo ajustado, mostraram tendência de associação na análise bivariada (tabela 3). Pessoas com ensino fundamental incompleto apresentaram a maior RP ajustada, de 1,39 (IC_95_% 0,91;2,12), e pessoas autodeclaradas pardas apresentaram a maior RP ajustada, de 1,33 (IC_95_% 0,97;1,81). A categoria “Indígena” não foi inserida na análise multivariável, devido à distribuição igual de 50% entre os grupos, o que impede a estimação precisa dos efeitos estatísticos.

**Tabela 3:** Razão de prevalência bruta e ajustada (RP) com intervalos de confiança de 95% (IC95%), do uso de medicamento psiquiátrico, de acordo com variáveis independentes, em pessoas do Ambulatório Trans, Porto Alegre, 2021-2022 (n = 629)

Variável	RP bruta (IC_95%_)	RP ajustada (IC_95%_)
Identidade de gênero		
Homem trans	1	1
Mulher trans e travesti	1,19 (0,90;1,57)	1,04 (0,76;1,40)
Não binário	1,11 (0,76;1,60)	1,23 (0,82;1,86)
Outros	1,47 (0,82;2,63)	1,05 (0,55;1,99)
**Faixa etária (anos)**		
18-23	1	1
24-29	0,99 (0,71;1,40)	0,96 (0,68;1,35)
30-39	1,15 (0,80;1,63)	1,10 (0,78;1,55)
40-49	2,37 (1,76;3,19)	2,06 (1,55;2,73)
50+	1,36 (0,59;3,11)	1,58 (0,78;3,19)
**Escolaridade**		
Ensino médio completo ou mais	1	1
Ensino médio incompleto	1,06 (0,76;1,46)	1,11 (0,80;1,54)
Ensino fundamental incompleto	1,68 (1,08;2,60)	1,39 (0,91;2,12)
**Raça/cor da pele**		
Branca	1	1
Parda	1,61 (1,18;2,19)	1.33 (0.97;1.81)
Preta	1,21 (0,86;1,69)	1.02 (0.72;1.45)
Amarela	0,53 (0,09;3,27)	0.49 (0.09;2.62)
**Retificação do nome social**		
Sim	1	1
Não	0,97 (0,70;1,33)	1,05 (0,76;1,45)
**Trabalho formal**		
Sim	1	1
Não	1,13 (0,84;1,51)	1,12 (0,83;1,52)
**Acesso a unidade de saúde**		
Sim	1	1
Não	0,45 (0,34;0,59)	0,46 (0,35;0,60)

## DISCUSSÃO 

Este estudo apresentou resultados que apontaram uma prevalência de dispensação de medicamentos psiquiátricos em 29% da amostra. Foram observados, enquanto fatores de risco na análise bivariada, ser pessoa de raça/cor da pele negra e ter ensino fundamental incompleto. Ademais, permaneceram associadas, no modelo ajustado, características como ser pessoa adulta com idade entre 40 e 49 anos e que teve acesso a unidade da APS. 

É importante ressaltar a falta de dados oficiais sobre pessoas trans e travestis no Brasil, um problema grave que dificulta a implementação e a qualificação de políticas públicas. No Censo e na Política Nacional de Saúde (PNS), não há campos relacionados à identidade de gênero. Assim, os dados disponíveis no Brasil são obtidos principalmente por meio de pesquisas acadêmicas e levantamentos realizados por organizações não governamentais coordenadas por pessoas trans, como a Antra.^5^

De acordo com a Organização Mundial da Saúde, a prevalência de transtornos mentais na população mundial é de 13%; entretanto, essa ocorrência pode variar de acordo com as diferentes características populacionais.^9^ No Brasil, não encontramos na literatura uma estimativa abrangente para todos os transtornos mentais comuns. No entanto, segundo a Pesquisa Nacional de Saúde de 2019, aproximadamente 10% da população relatou ter recebido diagnóstico de depressão por um profissional de saúde mental.^10^ Em relação ao uso de medicamentos psiquiátricos, encontramos apenas uma revisão sistemática que aborda a população brasileira como um todo. No entanto, esta revisão se limita a discutir a prevalência de uso de uma classe específica de medicamentos, os antidepressivos, que apresentou uma proporção geral de 4% nos estudos avaliados.^11^ Em nosso estudo, encontramos uma frequência de 31,5% para fluoxetina e 10,7% para amitriptilina. Considerando-se que as pessoas transsexuais e travestis são mais expostas a violências diversas e estão sujeitas a maior vulnerabilidade social, o risco de desenvolvimento de problemas de saúde mental se mostra maior em comparação com a população geral. Isso se verifica mesmo entre as pessoas trans, variando a partir de fatores como idade, raça/cor da pele, local de moradia, escolaridade e situação econômica.^12,13^

Em relação à mediana de idade no estudo para dispensação de medicação psiquiátrica, observou-se que pessoas de maior idade apresentaram maior prevalência na retirada de medicamentos, bem como pessoas de 40 a 49 anos mostraram maior RP Ajustada. Esse fenômeno pode ser explicado, em parte, pelo fato de terem crescido e vivenciado seus corpos em uma sociedade que considerava a orientação sexual e a identidade de gênero como transtornos mentais.^14^ Assim, somente em 2019, a transexualidade foi deslocada do grupo de transtorno de saúde mental para integrar o grupo de condições relacionadas à saúde sexual, na Classificação Internacional de Doenças. Estudos apontam que a de despatologização das identidades trans, os processos de afirmação de gênero, o uso do nome social e do gênero autodeclarado foram associados à redução dos sintomas de ansiedade e depressão, resultando no sentimento de pertencimento, melhora da integração social, maior vinculação das pessoas trans aos serviços de saúde e melhora na qualidade de vida.^15,16^

A dispensação de medicamentos psiquiátricos foi mais prevalente em pessoas autodeclaradas pretas e pardas, com menor grau de escolaridade, apesar de ser observado o maior acesso ao ambulatório por pessoas brancas e altamente escolarizadas. Diante disso, estudos mostram que pessoas trans autodeclaradas negras, que sofrem mais com a discriminação e a violência, apresentam um nível de escolaridade mais baixo, maiores chances de estarem em subempregos, piores condições socioeconômicas e menor expectativa de vida, resultando em um risco significativamente maior de desenvolver problemas de saúde e níveis elevados de sofrimento psicológico.^17,18^ Conforme levantamento da Antra, entre 2017 e 2023, cerca de 78,7% das pessoas trans assassinadas eram negras e 57% eram profissionais do sexo.^5^ Frente a isso, o conceito da interseccionalidade permite compreender melhor esse resultado, pois analisa as múltiplas formas de discriminação que uma pessoa pode sofrer, as quais acometem de forma mais acentuada pessoas trans, negras e pertencentes a classes sociais de menor poder aquisitivo.^18^

Em relação ao acesso à saúde, reconhecendo a vulnerabilidade da população trans e seu histórico distanciamento do SUS, a PNSILGBT foi lançada, para garantir o acesso universal à saúde dessa população. Ela estabelece diretrizes para acolhimento e cuidado integral, prevenção e promoção da saúde. Entretanto, na tentativa de acessar os serviços de saúde, as pessoas trans se deparam com diversas barreiras, como a patologização das suas identidades, o preconceito institucional e a discriminação dos profissionais de saúde, a resistência da utilização do nome social e do pronome adequado, e a falta de acolhimento.^19,20^ Essas barreiras contribuem para a demora na busca por tratamentos preventivos, aumentando o risco de desenvolver doenças crônicas e o agravamento de problemas de saúde mental. 

A discriminação, imposta historicamente pela sociedade heteronormativa, sexista, racista e classista, pode levar a população trans à marginalização social e institucional, contribuindo para o aumento do risco de desenvolvimento de problemas de saúde mental, comportamento de risco para infecções sexualmente transmissíveis e tendência ao abuso de substâncias lícitas e ilícitas.^4,6,7^ Neste estudo, medicamentos que apresentaram prevalência na dispensação foram fluoxetina, seguido da clorpromazina, carbonato de lítio, amitriptilina e ácido valpróico. Apesar de apresentarmos a frequência de medicamentos dispensados segundo a classificação da ATC, utilizamos a perspectiva da abordagem e indicação terapêutica do uso dos medicamentos na discussão, para ampliar o sentido do uso terapêutico no cuidado em saúde mental. 

Em geral, os estudos indicam alta prevalência de transtornos mentais, como depressão, ansiedade e suicídio em pessoas trans, em decorrência das violências de natureza transfóbica sofridas cotidianamente.^21^ Os dados trazidos nesta pesquisa corroboram a literatura científica no que tange ao sofrimento dessa parcela da população: cerca de 70% das 659 pessoas que compõem o estudo apresentam diagnóstico de depressão e ansiedade, além de uma parcela significativa ter relatado pensamentos de morte.^22^ Além disso, apresentaram maiores chances de receber prescrições de antidepressivos e ansiolíticos e de serem hospitalizadas após uma tentativa de suicídio.^23^ Em nosso estudo, observamos prevalência na dispensação de fluoxetina, amitriptilina, clonazepam e diazepam, consideradas medicamentos de escolha no tratamento de depressão e ansiedade, reduzindo os sintomas.

Podemos associar as disparidades em saúde mental das pessoas trans ao estresse de minoria, pois os estressores cotidianos e os estressores específicos impactam a piora dos indicadores de saúde mental e qualidade de vida dessa população.^24^ Um estudo realizado em Chicago, que utilizou uma coorte populacional de jovens de minorias sexuais e de gênero, associou a rejeição familiar, entre os transgêneros, a maiores sintomas de depressão e isolamento social associado à ideação suicida. O mesmo estudo comparou o suporte social, familiar e de amigos, entre jovens trans e jovens cisgêneros de minoria sexual, e identificou que jovens trans teriam menor suporte dentro de suas relações.^25^ Essa descoberta vem ao encontro de outras pesquisas que obtiveram uma forte correlação entre estigmatização e discriminação associadas à depressão grave.^26^

A literatura, também, sugere que a carga excessiva de estressores sociais associados ao baixo nível de escolarização, o acesso informal ao mercado de trabalho e o suporte social fragilizado está vinculado ao uso precoce de substâncias psicoativas e maior risco de abuso dessas substâncias.^27,28^ Ademais, é importante ressaltar que o sofrimento mental não se dá apenas pela identidade de gênero, mas pela intersecção entre gênero, raça, território e nível educacional, com parcelas específicas dessa população (pessoas negras e empobrecidas) sendo expostas a mais riscos em relação a sua sobrevivência.^5^

Em nosso estudo, os resultados da RP do uso de medicação psiquiátrica colaboram para a discussão no campo das políticas de saúde da população trans. Saliente-se que, apesar dos esforços representados pela instituição da PNSILGBT, ainda se tem um desafio para a garantia de que a população acesse de forma equânime a saúde. Diante dos resultados identificados neste estudo, podemos asseverar que as desigualdades de acesso aos serviços de saúde intensificam as vulnerabilidades e desigualdades históricas vivenciadas por essas pessoas. As pessoas trans não experimentam sofrimento por serem trans em si, mas devido à falta de aceitação e ao preconceito presente na sociedade, que contribui negativamente, resultando em desafios nas mais diversas áreas da vida, como educação, trabalho, relacionamentos e saúde.

É importante destacar que o tratamento medicamentoso, quando administrado de maneira segura e apropriada, pode controlar e aliviar os sintomas dos transtornos mentais. No entanto, o uso de medicamentos psiquiátricos enfrenta estigmas, devido a concepções equivocadas sobre os transtornos mentais e suas terapias. A decisão de prescrever psicofármacos deve considerar o diagnóstico do paciente, seu histórico de saúde, idade, comorbidades e uso concomitante de outros medicamentos ou substâncias. Se, por um lado, discutimos se a prevalência do uso de medicamentos psiquiátricos pode ser um intermediário para a alta ocorrência de transtornos mentais na população trans, por outro lado, reconhecemos a importância da equidade e da garantia do acesso a tecnologias farmacológicas.

O acesso aos medicamentos deve ser visto como uma parte essencial do cuidado em saúde mental, especialmente se considerando que a população trans enfrenta diversos impedimentos para utilizar serviços de saúde. A falta de serviços de saúde mental disponíveis no SUS agrava essa situação, resultando em um acesso reduzido a medicamentos psiquiátricos e psicoterapias para esse grupo. Portanto, melhorar o acesso seguro e adequado a medicamentos é crucial para atender às necessidades de saúde mental dessas populações vulneráveis.

Além disso, ao acessar os serviços de saúde, as pessoas trans se deparam com barreiras organizacionais complexas e multifacetadas que estão relacionadas a discriminação, patologização, acolhimento inadequado e a fatores sociodemográficos, o que resulta na evasão dos cuidados.^29^ É preciso considerar que o sucesso para um modelo de saúde igualitário, e que contemple o cuidado integral, pressupõe o reconhecimento às especificidades de cada grupo. Uma estratégia para romper com essas barreiras e haver a implementação plena da política inclui a formação dos profissionais de saúde e de apoio, para aperfeiçoar o acolhimento e atendimento dessa população.^30^

As limitações deste estudo devem-se ao seu delineamento transversal, que impossibilita a realização de inferências sobre mudanças de exposições ao longo do tempo. Além disso, a escassez de estudos que abordam o uso de medicamentos na população trans destaca a necessidade de mais pesquisas sobre o tema, para que políticas públicas efetivas possam ser reestruturadas, reconhecendo-se que a alta prevalência do uso de medicamentos pode refletir o sofrimento mental devido às iniquidades na saúde. Acreditamos que a frequência do uso de medicamentos foi subestimada, pois utilizamos apenas a dispensação como instrumento de coleta secundário. Para tanto, o uso de escalas psicométricas, por meio de questionários validados, deve ser encorajado. Outro fator limitante é o problema do acesso ao tratamento e, consequentemente, aos medicamentos, o que pode ter contribuído para a subestimação do problema. 

Este estudo estimou a prevalência de dispensação de medicamentos psiquiátricos e os fatores associados em pessoas atendidas no Ambulatório Trans de uma capital do Sul do Brasil. Os resultados sinalizam que 29% das pessoas cadastradas no serviço fizeram a retirada de uma ou mais medicamentos usados para tratamento de transtornos mentais, sendo o quesito idade e a falta de acesso a unidade da APS fatores associados ao desfecho. Escolaridade e raça/cor da pele, apesar de não permanecerem no modelo ajustado, apresentaram medidas de médio efeito na análise ajustada. Discutimos, a partir dos achados, que o estigma e a discriminação são fatores de vulnerabilidade que expõem a população trans a níveis elevados de sofrimento mental, sendo este aumentado se associado a gênero, raça/cor da pele, escolaridade e classe social.
